# 2-({[4-(1,3-Benzothia­zol-2-yl)phen­yl]amino}methyl)­phenol

**DOI:** 10.1107/S1600536811051580

**Published:** 2011-12-03

**Authors:** Kim Potgieter, Thomas Gerber, Eric Hosten, Richard Betz

**Affiliations:** a Nelson Mandela Metropolitan University, Summerstrand Campus, Department of Chemistry, University Way, Summerstrand, PO Box 77000, Port Elizabeth 6031, South Africa

## Abstract

In the title compound, C_20_H_16_N_2_OS, the aniline substituent essentially coplanar with the benzothia­zole moiety (with an r.m.s. deviation of all fitted non-H atoms of 0.0612 Å). The phenol group is almost perpendic­ular to the benzothia­zolylaniline group, with an inter­planar angle of 88.36 (2)°. In the crystal, mol­ecules aggregate as centrosymmetric dimers by pairs of O—H⋯N hydrogen bonds. C—H⋯O contacts and N—H⋯π(arene) inter­actions also occur.

## Related literature

For general information about rhenium-supported radio-pharmaceuticals, see: Gerber *et al.* (2011 ▶). For the crystal structure of 4-(1,3-benzothia­zol-2-yl)-*N*-(2-pyridyl­meth­yl)aniline monohydrate, see: Su *et al.* (2009 ▶). For graph-set analysis of hydrogen bonds, see: Etter *et al.* (1990 ▶); Bernstein *et al.* (1995 ▶).
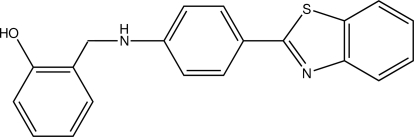


## Experimental

### 

#### Crystal data

C_20_H_16_N_2_OS
*M* *_r_* = 332.41
Monoclinic, 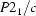

*a* = 13.3260 (4) Å
*b* = 5.7940 (1) Å
*c* = 24.2246 (6) Å
β = 121.546 (1)°
*V* = 1593.99 (7) Å^3^
*Z* = 4
Mo *K*α radiation
μ = 0.21 mm^−1^
*T* = 200 K
0.44 × 0.17 × 0.11 mm

#### Data collection

Bruker APEXII CCD diffractometer
Absorption correction: multi-scan (*SADABS*; Bruker, 2008 ▶) *T*_min_ = 0.929, *T*_max_ = 1.000
15126 measured reflections
3953 independent reflections
3272 reflections with *I* > 2σ(*I*)
*R*_int_ = 0.018

#### Refinement

*R*[*F* ^2^ > 2σ(*F* ^2^)] = 0.033
*wR*(*F* ^2^) = 0.092
*S* = 1.03
3953 reflections
221 parameters
H atoms treated by a mixture of independent and constrained refinement
Δρ_max_ = 0.30 e Å^−3^
Δρ_min_ = −0.23 e Å^−3^

### 

Data collection: *APEX2* (Bruker, 2010 ▶); cell refinement: *SAINT* (Bruker, 2010 ▶); data reduction: *SAINT*; program(s) used to solve structure: *SIR97* (Altomare *et al.*, 1999 ▶); program(s) used to refine structure: *SHELXL97* (Sheldrick, 2008 ▶); molecular graphics: *ORTEP-3* (Farrugia, 1997 ▶) and *Mercury* (Macrae *et al.*, 2008 ▶); software used to prepare material for publication: *SHELXL97* and *PLATON* (Spek, 2009 ▶).

## Supplementary Material

Crystal structure: contains datablock(s) I, global. DOI: 10.1107/S1600536811051580/gg2063sup1.cif

Supplementary material file. DOI: 10.1107/S1600536811051580/gg2063Isup2.cdx

Structure factors: contains datablock(s) I. DOI: 10.1107/S1600536811051580/gg2063Isup3.hkl

Supplementary material file. DOI: 10.1107/S1600536811051580/gg2063Isup4.cml

Additional supplementary materials:  crystallographic information; 3D view; checkCIF report

## Figures and Tables

**Table 1:** Hydrogen-bond geometry (Å, °) *Cg* is the centroid of the C31–C36 ring.

*D*—H⋯*A*	*D*—H	H⋯*A*	*D*⋯*A*	*D*—H⋯*A*
O1—H1⋯N1^i^	0.82	1.95	2.7459 (14)	164
C26—H26⋯O1^i^	0.95	2.48	3.3645 (16)	156
N2—H72⋯*Cg*^ii^	0.82 (2)	2.61 (2)	3.4024 (14)	163.0 (19)

Symmetry codes: (i) 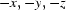; (ii) 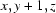.

**Fig. 1.**
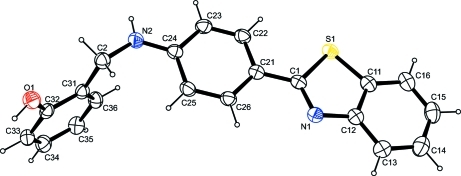
The molecular structure of the title compound, with atom labels and anisotropic displacement ellipsoids (drawn at 50% probability level).

**Fig. 2.**
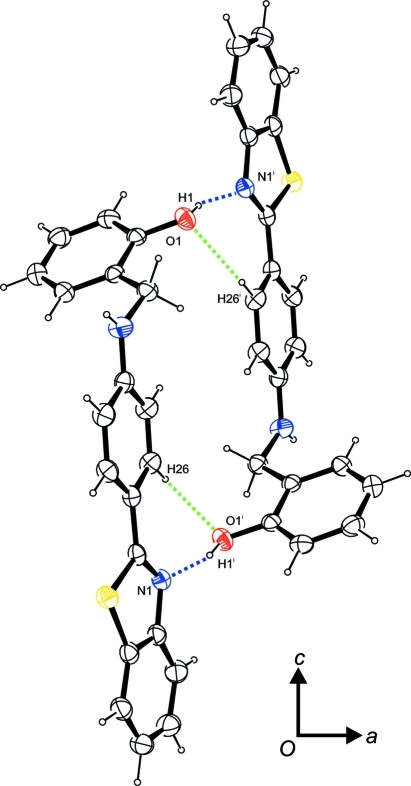
Intermolecular contacts, viewed along [0 1 0]. Blue dashed lines indicate classical hydrogen bonds of the O–H···N type, green dashed lines indicate C–H···O contacts. Symmetry operator: i -x, -y, -z.

**Fig. 3.**
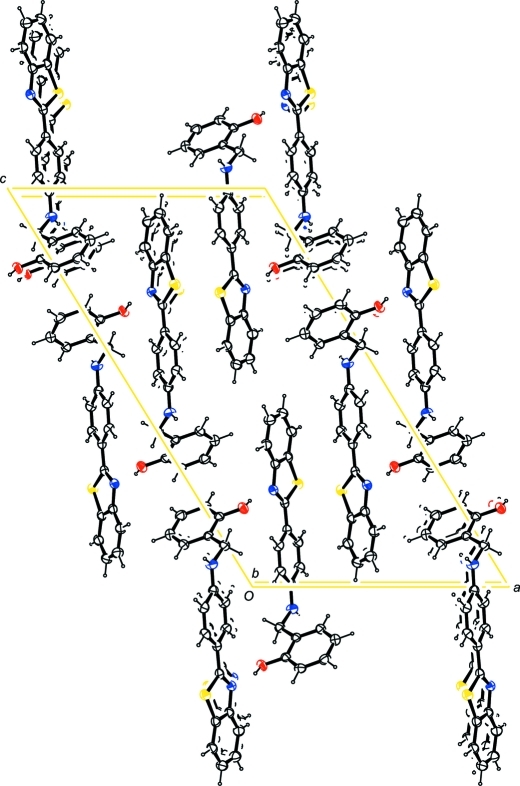
Molecular packing of the title compound, viewed along [0 1 0] (anisotropic displacement ellipsoids drawn at 50% probability level).

## supplementary crystallographic information

### Comment

In our continuous efforts to create new radio-pharmaceuticals (Gerber *et al.*, 2011), we attempted the coordination reaction of a potentially multidentate ligand towards a rhenium precursor upon which a crystalline reaction product was obtained. The crystal structure analysis showed the presence of the free ligand only whose molecular and crystal structure has not been reported to date. The structure of 4-(1,3-benzothiazol-2-yl)-*N*-(2-pyridylmethyl) aniline monohydrate is noted in the literature (Su *et al.*, 2009).

The benzothiazolyl system and the attached aniline system are nearly co-planar (r.m.s. of all fitted non-hydrogen atoms including the nitrogen bound methylene group = 0.0612 Å). The phenolic substituent, however, adopts a nearly perpendicular orientation with respect to the rest of the molecule, with an interplanar angle of 88.36 (2)° between the two least-squares planes defined by both moieties (Fig. 1).

In the crystal, classical hydrogen bonds of the O–H···N type as well as C–H···O contacts (whose range lies by more than 0.1 Å below the sum of van-der-Waals radii of the atoms participating) are observed. The latter are supported by one of the hydrogen atoms of the central phenyl ring. In total, the molecules are connected to centrosymmetric dimers by these two interactions. In terms of graph-set analysis (Etter *et al.*, 1990; Bernstein *et al.*, 1995), the descriptor for these contacts is *R*^2^_2_(18)*R*^2^_2_(24) on the unitary level. The nitrogen-bonded hydrogen atom forms a hydrogen bond to the aromatic system of the phenolic moiety, connecting the molecules to chains along the crystallographic *b* axis. Metrical parameters about these contacts as well as information about their symmetry is listed in Table 1. The shortest intercentroid distance between two aromatic systems was measured at 4.6019 (10) Å and is apparent between the phenyl unit of the benzothiazole moiety and the central C_6_ aromatic ring (Fig 2.)

The packing of the title compound in the crystal structure is shown in Figure 3.

### Experimental

A mixture of 2.00 g of 4-aminobenzoic acid and 1.33 g of 2-aminothiophenol was added to hot polyphosphoric acid. The stirring solution was heated to 220 °C for four hours. The reaction solution was cooled to room temperature and poured into a 10% K_2_CO_3_ solution. The yellow precipitate which formed was filtered and dried under vacuum, yielding 4-(benzo[*d*]thiazol-2-yl)benzenamine. A solution of 1.0 g of this product dissolved in 25 cm^3^ of methanol was added to a 25 cm^3^ methanol solution of 2-hydroxybenzaldehyde (0.4 g). The solution was refluxed for three hours after which it was cooled to room temperature and stirred overnight. An excess of NaBH_4_ (2.0 g) was added in portions with stirring and the mixture was left to stir at room temperature overnight. The solvent was removed by evaporation and 50 cm^3^ of water was added. HCl was added to adjust the pH to 6, resulting in the formation of a light yellow precipitate which was filtered and dried under vacuum. Crystals suitable for the X-ray diffraction study were obtained upon the attempted synthesis of a rhenium coordination compound in ethanol.

### Refinement

Carbon-bound H atoms were placed in calculated positions (C—H 0.95 Å for aromatic carbon atoms, C—H 0.99 Å for the methylene group) and were included in the refinement in the riding model approximation, with *U*(H) set to 1.2*U*_eq_(C). The H atom of the hydroxyl group was allowed to rotate with a fixed angle around the C—O bond to best fit the experimental electron density (HFIX 147 in the *SHELX* program suite (Sheldrick, 2008)), with *U*(H) set to 1.5*U*_eq_(O). The nitrogen-bound H atom was located on a difference Fourier map and refined freely with isotropic parameters.

### Crystal data

**Table:**

C_20_H_16_N_2_OS	*F*(000) = 696
*M**_r_* = 332.41	*D*_x_ = 1.385 Mg m^−^^3^
Monoclinic, *P*2_1_/*c*	Mo *K*α radiation, λ = 0.71069 Å
Hall symbol: -P 2ybc	Cell parameters from 7469 reflections
*a* = 13.3260 (4) Å	θ = 3.1–28.3°
*b* = 5.7940 (1) Å	µ = 0.21 mm^−^^1^
*c* = 24.2246 (6) Å	*T* = 200 K
β = 121.546 (1)°	Platelet, brown
*V* = 1593.99 (7) Å^3^	0.44 × 0.17 × 0.11 mm
*Z* = 4	

### Data collection

**Table:**

Bruker APEXII CCD diffractometer	3953 independent reflections
Radiation source: fine-focus sealed tube	3272 reflections with *I* > 2σ(*I*)
graphite	*R*_int_ = 0.018
φ and ω scans	θ_max_ = 28.3°, θ_min_ = 1.9°
Absorption correction: multi-scan (*SADABS*; Bruker, 2008)	*h* = −17→17
*T*_min_ = 0.929, *T*_max_ = 1.000	*k* = −4→7
15126 measured reflections	*l* = −31→32

### Refinement

**Table:**

Refinement on *F*^2^	Primary atom site location: structure-invariant direct methods
Least-squares matrix: full	Secondary atom site location: difference Fourier map
*R*[*F*^2^ > 2σ(*F*^2^)] = 0.033	Hydrogen site location: inferred from neighbouring sites
*wR*(*F*^2^) = 0.092	H atoms treated by a mixture of independent and constrained refinement
*S* = 1.03	*w* = 1/[σ^2^(*F*_o_^2^) + (0.0417*P*)^2^ + 0.6757*P*] where *P* = (*F*_o_^2^ + 2*F*_c_^2^)/3
3953 reflections	(Δ/σ)_max_ = 0.001
221 parameters	Δρ_max_ = 0.30 e Å^−^^3^
0 restraints	Δρ_min_ = −0.23 e Å^−^^3^

### Fractional atomic coordinates and isotropic or equivalent isotropic displacement parameters (Å^2^)

**Table:**

	*x*	*y*	*z*	*U*_iso_*/*U*_eq_	
S1	0.42147 (3)	0.68632 (6)	0.257240 (17)	0.03011 (10)	
O1	−0.15303 (8)	0.05617 (17)	−0.19880 (5)	0.0317 (2)	
H1	−0.1951	−0.0563	−0.2167	0.048*	
N1	0.31149 (9)	0.30173 (19)	0.24291 (5)	0.0264 (2)	
N2	0.09494 (11)	0.5026 (2)	−0.06249 (6)	0.0317 (3)	
H72	0.1055 (16)	0.621 (4)	−0.0774 (9)	0.049 (5)*	
C1	0.32433 (11)	0.4656 (2)	0.20995 (6)	0.0246 (3)	
C2	0.00373 (12)	0.3473 (2)	−0.10753 (6)	0.0291 (3)	
H2A	−0.0541	0.4367	−0.1460	0.035*	
H2B	−0.0380	0.2851	−0.0869	0.035*	
C11	0.44852 (11)	0.5440 (2)	0.32639 (7)	0.0284 (3)	
C12	0.38217 (11)	0.3396 (2)	0.30904 (6)	0.0273 (3)	
C13	0.39226 (13)	0.1916 (3)	0.35715 (7)	0.0343 (3)	
H13	0.3484	0.0521	0.3461	0.041*	
C14	0.46741 (14)	0.2528 (3)	0.42110 (7)	0.0398 (3)	
H14	0.4755	0.1534	0.4544	0.048*	
C15	0.53187 (13)	0.4580 (3)	0.43785 (7)	0.0390 (3)	
H15	0.5821	0.4962	0.4823	0.047*	
C16	0.52407 (12)	0.6063 (3)	0.39127 (7)	0.0344 (3)	
H16	0.5683	0.7454	0.4028	0.041*	
C21	0.26744 (11)	0.4708 (2)	0.13979 (6)	0.0254 (3)	
C22	0.27965 (12)	0.6609 (2)	0.10797 (7)	0.0295 (3)	
H22	0.3280	0.7866	0.1328	0.035*	
C23	0.22327 (12)	0.6691 (2)	0.04174 (7)	0.0305 (3)	
H23	0.2331	0.8004	0.0215	0.037*	
C24	0.15098 (11)	0.4861 (2)	0.00317 (6)	0.0266 (3)	
C25	0.14071 (12)	0.2928 (2)	0.03483 (7)	0.0296 (3)	
H25	0.0941	0.1652	0.0101	0.035*	
C26	0.19777 (12)	0.2865 (2)	0.10154 (6)	0.0286 (3)	
H26	0.1896	0.1541	0.1220	0.034*	
C31	0.04626 (11)	0.1472 (2)	−0.12987 (6)	0.0250 (3)	
C32	−0.03897 (11)	0.0004 (2)	−0.17724 (6)	0.0258 (3)	
C33	−0.00650 (12)	−0.1827 (2)	−0.20165 (6)	0.0306 (3)	
H33	−0.0649	−0.2806	−0.2340	0.037*	
C34	0.11211 (13)	−0.2216 (3)	−0.17839 (7)	0.0351 (3)	
H34	0.1348	−0.3467	−0.1949	0.042*	
C35	0.19715 (12)	−0.0793 (3)	−0.13149 (7)	0.0343 (3)	
H35	0.2781	−0.1073	−0.1155	0.041*	
C36	0.16400 (11)	0.1046 (3)	−0.10779 (6)	0.0302 (3)	
H36	0.2228	0.2031	−0.0759	0.036*	

### Atomic displacement parameters (Å^2^)

**Table:**

	*U*^11^	*U*^22^	*U*^33^	*U*^12^	*U*^13^	*U*^23^
S1	0.02935 (17)	0.02586 (17)	0.03296 (18)	−0.00579 (12)	0.01480 (14)	−0.00429 (13)
O1	0.0241 (5)	0.0321 (5)	0.0340 (5)	−0.0054 (4)	0.0118 (4)	−0.0005 (4)
N1	0.0234 (5)	0.0264 (5)	0.0283 (5)	−0.0015 (4)	0.0129 (4)	−0.0011 (4)
N2	0.0387 (7)	0.0273 (6)	0.0282 (6)	−0.0073 (5)	0.0170 (5)	−0.0016 (5)
C1	0.0209 (6)	0.0219 (6)	0.0310 (6)	−0.0003 (4)	0.0137 (5)	−0.0028 (5)
C2	0.0284 (6)	0.0303 (7)	0.0277 (6)	−0.0025 (5)	0.0139 (5)	−0.0015 (5)
C11	0.0235 (6)	0.0298 (7)	0.0322 (7)	0.0015 (5)	0.0147 (5)	−0.0033 (5)
C12	0.0227 (6)	0.0293 (6)	0.0297 (6)	0.0017 (5)	0.0137 (5)	−0.0015 (5)
C13	0.0342 (7)	0.0341 (7)	0.0349 (7)	0.0010 (6)	0.0182 (6)	0.0023 (6)
C14	0.0390 (8)	0.0489 (9)	0.0314 (7)	0.0077 (7)	0.0184 (6)	0.0055 (7)
C15	0.0305 (7)	0.0531 (9)	0.0281 (7)	0.0051 (6)	0.0117 (6)	−0.0059 (6)
C16	0.0264 (6)	0.0396 (8)	0.0342 (7)	0.0003 (6)	0.0138 (6)	−0.0093 (6)
C21	0.0231 (6)	0.0245 (6)	0.0300 (6)	0.0003 (5)	0.0149 (5)	−0.0010 (5)
C22	0.0281 (6)	0.0244 (6)	0.0332 (7)	−0.0057 (5)	0.0142 (6)	−0.0027 (5)
C23	0.0328 (7)	0.0252 (6)	0.0339 (7)	−0.0044 (5)	0.0177 (6)	0.0018 (5)
C24	0.0275 (6)	0.0246 (6)	0.0299 (6)	0.0004 (5)	0.0166 (5)	−0.0007 (5)
C25	0.0352 (7)	0.0237 (6)	0.0314 (7)	−0.0058 (5)	0.0186 (6)	−0.0043 (5)
C26	0.0346 (7)	0.0228 (6)	0.0321 (7)	−0.0030 (5)	0.0201 (6)	−0.0009 (5)
C31	0.0266 (6)	0.0275 (6)	0.0216 (6)	−0.0027 (5)	0.0131 (5)	0.0017 (5)
C32	0.0253 (6)	0.0294 (6)	0.0211 (6)	−0.0027 (5)	0.0111 (5)	0.0038 (5)
C33	0.0344 (7)	0.0301 (7)	0.0236 (6)	−0.0045 (5)	0.0128 (5)	−0.0019 (5)
C34	0.0400 (8)	0.0370 (8)	0.0309 (7)	0.0022 (6)	0.0204 (6)	−0.0031 (6)
C35	0.0279 (7)	0.0420 (8)	0.0337 (7)	0.0009 (6)	0.0165 (6)	−0.0007 (6)
C36	0.0260 (6)	0.0350 (7)	0.0274 (6)	−0.0046 (5)	0.0124 (5)	−0.0023 (5)

### Geometric parameters (Å, °)

**Table:**

S1—C11	1.7275 (14)	C16—H16	0.9500
S1—C1	1.7526 (13)	C21—C26	1.3999 (18)
O1—C32	1.3627 (15)	C21—C22	1.4018 (18)
O1—H1	0.8200	C22—C23	1.3709 (19)
N1—C1	1.3082 (17)	C22—H22	0.9500
N1—C12	1.3866 (16)	C23—C24	1.4083 (18)
N2—C24	1.3616 (17)	C23—H23	0.9500
N2—C2	1.4459 (17)	C24—C25	1.4038 (18)
N2—H72	0.82 (2)	C25—C26	1.3804 (19)
C1—C21	1.4547 (18)	C25—H25	0.9500
C2—C31	1.5098 (19)	C26—H26	0.9500
C2—H2A	0.9900	C31—C36	1.3893 (18)
C2—H2B	0.9900	C31—C32	1.4022 (17)
C11—C16	1.3995 (19)	C32—C33	1.3890 (19)
C11—C12	1.4042 (19)	C33—C34	1.391 (2)
C12—C13	1.3954 (19)	C33—H33	0.9500
C13—C14	1.380 (2)	C34—C35	1.381 (2)
C13—H13	0.9500	C34—H34	0.9500
C14—C15	1.397 (2)	C35—C36	1.388 (2)
C14—H14	0.9500	C35—H35	0.9500
C15—C16	1.378 (2)	C36—H36	0.9500
C15—H15	0.9500		
			
C11—S1—C1	89.62 (6)	C22—C21—C1	121.28 (11)
C32—O1—H1	109.4	C23—C22—C21	121.38 (12)
C1—N1—C12	111.32 (11)	C23—C22—H22	119.3
C24—N2—C2	124.65 (12)	C21—C22—H22	119.3
C24—N2—H72	117.2 (13)	C22—C23—C24	121.07 (12)
C2—N2—H72	117.3 (13)	C22—C23—H23	119.5
N1—C1—C21	124.99 (11)	C24—C23—H23	119.5
N1—C1—S1	114.74 (10)	N2—C24—C25	122.93 (12)
C21—C1—S1	120.26 (9)	N2—C24—C23	119.26 (12)
N2—C2—C31	115.06 (11)	C25—C24—C23	117.81 (12)
N2—C2—H2A	108.5	C26—C25—C24	120.60 (12)
C31—C2—H2A	108.5	C26—C25—H25	119.7
N2—C2—H2B	108.5	C24—C25—H25	119.7
C31—C2—H2B	108.5	C25—C26—C21	121.54 (12)
H2A—C2—H2B	107.5	C25—C26—H26	119.2
C16—C11—C12	121.61 (13)	C21—C26—H26	119.2
C16—C11—S1	128.92 (11)	C36—C31—C32	118.40 (12)
C12—C11—S1	109.46 (10)	C36—C31—C2	123.94 (12)
N1—C12—C13	125.32 (12)	C32—C31—C2	117.63 (11)
N1—C12—C11	114.83 (12)	O1—C32—C33	123.46 (12)
C13—C12—C11	119.84 (13)	O1—C32—C31	115.67 (12)
C14—C13—C12	118.43 (14)	C33—C32—C31	120.80 (12)
C14—C13—H13	120.8	C32—C33—C34	119.49 (12)
C12—C13—H13	120.8	C32—C33—H33	120.3
C13—C14—C15	121.28 (15)	C34—C33—H33	120.3
C13—C14—H14	119.4	C35—C34—C33	120.40 (13)
C15—C14—H14	119.4	C35—C34—H34	119.8
C16—C15—C14	121.42 (14)	C33—C34—H34	119.8
C16—C15—H15	119.3	C34—C35—C36	119.79 (13)
C14—C15—H15	119.3	C34—C35—H35	120.1
C15—C16—C11	117.41 (14)	C36—C35—H35	120.1
C15—C16—H16	121.3	C35—C36—C31	121.11 (12)
C11—C16—H16	121.3	C35—C36—H36	119.4
C26—C21—C22	117.56 (12)	C31—C36—H36	119.4
C26—C21—C1	121.16 (11)		
			
C12—N1—C1—C21	176.61 (11)	C1—C21—C22—C23	−178.02 (12)
C12—N1—C1—S1	−1.79 (14)	C21—C22—C23—C24	−0.1 (2)
C11—S1—C1—N1	1.11 (10)	C2—N2—C24—C25	11.3 (2)
C11—S1—C1—C21	−177.37 (11)	C2—N2—C24—C23	−169.07 (13)
C24—N2—C2—C31	−93.43 (16)	C22—C23—C24—N2	178.90 (13)
C1—S1—C11—C16	178.94 (13)	C22—C23—C24—C25	−1.4 (2)
C1—S1—C11—C12	−0.09 (10)	N2—C24—C25—C26	−178.83 (13)
C1—N1—C12—C13	−177.24 (13)	C23—C24—C25—C26	1.5 (2)
C1—N1—C12—C11	1.72 (15)	C24—C25—C26—C21	−0.1 (2)
C16—C11—C12—N1	180.00 (12)	C22—C21—C26—C25	−1.5 (2)
S1—C11—C12—N1	−0.88 (14)	C1—C21—C26—C25	178.10 (12)
C16—C11—C12—C13	−1.0 (2)	N2—C2—C31—C36	2.59 (19)
S1—C11—C12—C13	178.14 (10)	N2—C2—C31—C32	−175.35 (11)
N1—C12—C13—C14	179.44 (13)	C36—C31—C32—O1	−177.51 (11)
C11—C12—C13—C14	0.5 (2)	C2—C31—C32—O1	0.55 (16)
C12—C13—C14—C15	0.3 (2)	C36—C31—C32—C33	−0.21 (18)
C13—C14—C15—C16	−0.8 (2)	C2—C31—C32—C33	177.85 (12)
C14—C15—C16—C11	0.3 (2)	O1—C32—C33—C34	177.58 (12)
C12—C11—C16—C15	0.5 (2)	C31—C32—C33—C34	0.49 (19)
S1—C11—C16—C15	−178.40 (11)	C32—C33—C34—C35	−0.1 (2)
N1—C1—C21—C26	−3.99 (19)	C33—C34—C35—C36	−0.5 (2)
S1—C1—C21—C26	174.33 (10)	C34—C35—C36—C31	0.8 (2)
N1—C1—C21—C22	175.56 (12)	C32—C31—C36—C35	−0.44 (19)
S1—C1—C21—C22	−6.12 (17)	C2—C31—C36—C35	−178.37 (13)
C26—C21—C22—C23	1.5 (2)		

### Hydrogen-bond geometry (Å, °)

**Table:**

*Cg* is the centroid of the C31–C36 ring.

**Table:**

*D*—H···*A*	*D*—H	H···*A*	*D*···*A*	*D*—H···*A*
O1—H1···N1^i^	0.82	1.95	2.7459 (14)	164.
C26—H26···O1^i^	0.95	2.48	3.3645 (16)	156.
N2—H72···*Cg*^ii^	0.82 (2)	2.61 (2)	3.4024 (14)	163.0 (19)

Symmetry codes: (i) −*x*, −*y*, −*z*; (ii) *x*, *y*+1, *z*.
